# ε-Poly-l-Lysine Enhances Fruit Disease Resistance in Postharvest Longans (*Dimocarpus longan* Lour.) by Modulating Energy Status and ATPase Activity

**DOI:** 10.3390/foods11050773

**Published:** 2022-03-07

**Authors:** Junzheng Sun, Hongbin Chen, Yihui Chen, Mengshi Lin, Yen-Con Hung, Yuji Jiang, Hetong Lin

**Affiliations:** 1 Institute of Postharvest Technology of Agricultural Products, College of Food Science, Fujian Agriculture and Forestry University, Fuzhou 350002, China; sunjzl@outlook.com (J.S.); yummyway@163.com (H.C.); jyj1209@163.com (Y.J.); hetonglin@163.com (H.L.); 2 College of Oceanology and Food Science, Quanzhou Normal University, Quanzhou 362000, China; 3 Key Laboratory of Postharvest Biology of Subtropical Special Agricultural Products, Fujian Province University, Fuzhou 350002, China; 4 Food Science Program, Division of Food, Nutrition & Exercise Sciences, University of Missouri, Columbia, MO 65211, USA; linme@missouri.edu; 5 Department of Food Science and Technology, University of Georgia, 1109 Experiment Street, Griffin, GA 30223, USA; yhung@uga.edu

**Keywords:** ε-poly-l-lysine (ε-PL), fruit disease resistance, energy status, adenosine triphosphatase (ATPase), longan fruit

## Abstract

ε-poly-l-lysine (ε-PL) holds a strong antibacterial property and is widely used for food preservation. However, the application of ε-PL to enhance fruit disease resistance in postharvest longans (*Dimocarpus longan* Lour.) has not been explored. The objective of this study was to explore the impact of ε-PL treatment on disease occurrence and energy metabolism of longans infected with *Phomopsis longanae* Chi (*P*. *longanae*). It was found that, in comparison with *P*. *longanae*-inoculated longans, ε-PL could decrease the fruit disease index and adenosine monophosphate (AMP) content, increase the amounts of adenosine triphosphate (ATP), adenosine diphosphate (ADP), and energy charge, and enhance the activities of adenosine triphosphatase (ATPase) (such as H^+^-, Mg^2+^-, and Ca^2+^-ATPase) in the mitochondria, protoplasm, and vacuole. The results suggest that the higher levels of ATPase activity and energy status played essential roles in disease resistance of postharvest longan fruit. Therefore, the ε-PL treatment can be used as a safe and efficient postharvest method to inhibit the disease occurrence of longan fruit during storage at room temperature.

## 1. Introduction

China ranks top in the world in terms of the planting area, the yield, and the varieties of longan (*Dimocarpus longan* Lour.) [1,2]. Longan fruit is hugely popular because of its high nutritional value, health benefits, and good taste. Longan is typically consumed in its fresh or dried form [3,4,5]. However, longan fruit ripens in hot and humid seasons and possesses vigorous respiratory metabolism after harvest, and it is susceptible to pathogenic fungi such as *Phomopsis longanae* Chi (*P. longanae*) [1]. *P. longanae* is a major pathogenic fungus that can cause disease in harvested longans [2,6,7]. However, many fungicides are not permitted for disease control on harvested fruits due to environmental concerns and their adverse effects on human health. Therefore, novel, safe, and efficient postharvest handling methods for disease control in longans are needed urgently.

Energy status is a crucial basis for keeping normal physiological metabolism in plants. To resist the further invasion of pathogens, defense reactions will occur in plants, which will consume much energy [8]. Many reports demonstrated that an increase in ATP and energy charge are related to the decrease in lesion development on peaches, pears, and muskmelons. The energy deficiency will accelerate the process of disease development [9,10,11]. Moreover, adenosine triphosphatase (ATPase) has a key effect on energy transportation, substance absorption, and ATP synthesis in the protoplasm, vacuole, and mitochondria. The level of ATPase activity can reflect the cell membranes integrity, which affects fruit disease resistance [8,12]. A previous study about strawberry fruit indicated that 2,4-dinitrophenol (DNP) could promote fruit disease development via decreasing energy charge and ATP content, keeping lower activities of H^+^-ATPase and Ca^2+^-ATPase, and finally destroying mitochondrial function, while the addition of ATP could slow this process [13]. Similar results were reported by Li et al. (2020) who indicated that benzothiadiazole treatment could greatly reduce the lesion diameter of *Penicillium expansum*-inoculated apple fruit and enhance H^+^- and Ca^2+^-ATPase activities, which was coincided with the raised ATP level and energy status [14]. Therefore, energy status and ATPase activities might play a central role in controlling fruit diseases.

ε-Poly-l-lysine (ε-PL), a natural fungicide isolated from *Streptomyces albulus* NO. 346, is composed of α-hydroxyl and ε-amino via peptide bond of L-lysine [15,16,17,18]. Unlike synthetic fungicides, ε-PL is safe for human beings and the environment. ε-PL has been extensively utilized for preserving food products in America, China, and Japan [15,19,20,21]. In vitro research showed that ε-PL could efficiently prevent the growth of *Alternaria alternata*, *Botrytis cinerea*, *Penicillium digitatum*, *Saccharomyces cerevisiae,* and *Staphylococcus aureus* [15,22,23,24,25]. ε-PL was able to inhibit fruit disease development in the strawberry, cherry tomato, grape, apple, jujube, and citrus fruit [23,25,26,27]. For example, it was reported that ε-PL addition could strengthen disease defense in the apple fruit against *Penicillium expansum* through activating the phenylpropanoid pathway and accumulating resistant substances such as flavonoids, lignin, and total phenolic compounds [27,28]. However, the influence of ε-PL treatment in *P*. *longanae*-inoculated longans on their disease resistance against *P*. *longanae* and its relationship with energy metabolism are still unknown. Therefore, this study aimed to examine the influence of ε-PL treatment on the fruit disease development, energy status (the contents of ADP, AMP and ATP, and energy charge), and ATPase activities (Ca^2+^-, H^+^-, and Mg^2+^-ATPase in membranes of protoplasm, vacuole, and mitochondria). This study also evaluated the availability of ε-PL as a safe and efficient postharvest handling for controlling longan fruit disease.

## 2. Materials and Methods

### 2.1. Materials and Treatments

‘Fuyan’ longan fruits were collected from a longan orchard (Nan’an, Fujian, China). The longan fruits were transported to the laboratory under 4 °C within 3 h. Fruit with uniformity (size, color, and shape) and without mechanical injury were selected. *P. longanae* was cultured and prepared in spore suspensions (1 × 10^4^ spores/L) based on an approach reported by Chen et al. (2014) [29].

ε-PL used in the study was analytically pure and purchased from Macklin Incorporated (Shanghai, China). In our preliminary tests, longan fruit were treated in 0, 50, 100, 150, 200, and 250 mg/L of ε-PL solutions and then inoculated with *P. longanae*, the lowest fruit disease index was 0.74 at day 5 with 150 mg/L ε-PL treatment. Therefore, 150 mg/L ε-PL was chosen for this study.

All fruit samples were divided into three groups (2000 fruit per group): (i) the control group, (ii) *P. longanae*-inoculated group, and (iii) ε-PL treatment group. The longan fruit in the control group and *P. longanae*-inoculated group were soaked in sterile distilled water (SDW) for 10 min and then air-dried. The longan fruit in the ε-PL treatment group were soaked in 150 mg/L of ε-PL solutions for 10 min and then air-dried. Next, the control fruit were soaked in SDW for 5 min, and the others were submerged in the suspension of *P. longanae* spore for 5 min. After treatment, the longan fruit were placed (50 fruits/bag) in polyethylene bags with a thickness of 0.015 mm, and transferred to a chamber with set conditions (temperature = 28 ± 1 °C; relative humidity = 90%), and stored for 5 days. Each day, six bags of each group were randomly withdrawn for the following analyses. Each experiment was performed in triplicate.

### 2.2. Measurement of Fruit Disease Index

The fruit disease index was analyzed based on a previous study, which was calculated as ∑(disease scale/the highest scale × proportion of corresponding fruit within each class). Visual appearance scale: 0, no lesion; 1, lesion area < 1/4; 2, 1/4 ≤ lesion area < 1/2; 3, 1/2 ≤ lesion area < 3/4; and 4, lesion area ≥ 3/4 [29]. Fifty longan fruits were sampled each day randomly for measuring the area proportion of the lesion on the surface.

### 2.3. Determination of ADP, AMP, ATP, and Energy Charge

Based on the protocols reported by Chen et al. (2018) and Zhang et al. (2019), 5 g of pericarp from 10 longan fruits were used to measure the contents of ADP, AMP, and ATP, and energy charge [30,31], which was conducted by a high-performance liquid chromatography (LC-2030C, Shimadzu Corporation, Kyoto, Japan). The quantification of ADP, AMP, and ATP was expressed as mg/kg. Energy charge was computed using a formula: (ATP + 1/2 ADP)/(ATP + ADP + AMP).

### 2.4. Measurement of ATPase Activity

The ATPase activity was determined according to the methods of previous studies [3,29]. One gram of pericarp selected from 10 longan fruits was used to extract three ATPases, including Ca^2+^-, H^+^-, and Mg^2+^-ATPase. To determine the activity of Ca^2+^-ATPase, 0.2 mL of reaction extract was mixed with 0.5 mL of reaction fluid containing 50 mmol/L of Tris-HCl (pH 7.5), 50 mmol/L of NaCl, 2 mmol/L of ethylene diamine tetraacetic acid (EDTA), 5 mmol/L of dithiothreitol (DTT), and 2 mmol/L of CaCl_2_. A reaction mixture was applied to analyze the activity of Mg^2+^-ATPase. The reaction mixture was composed of 0.2 mL reaction extract and 0.5 mL reaction fluid (50 mmol/L of NaCl, 50 mmol/L of Tris-HCl (pH 7.5), 2 mmol/L of EDTA, 5 mmol/L of DTT, and 5 mmol/L of MgCl_2_). For the measurement of H^+^-ATPase activities, the reaction mixture was utilized, including 0.2 mL reaction extract and 0.5 mL reaction fluid (50 mmol/L of Tris-HCl at pH 7.5, 0.5 mmol/L of KCl, and 20 mmol/L of MgSO_4_). The absorbance values were acquired at 660 nm. The results were presented with a unit of U/kg.

### 2.5. Statistical Analyses

All the data were determined thrice and recorded as the mean ± standard error (*n* = 3). The IBM SPSS Statistics (Version 21, New York, NY, USA) with T-test tool was used to verify the significant difference between the comparison of *P. longanae*-inoculated group and control group, ε-PL-treated *P. longanae*-inoculated group and *P. longanae*-inoculated group. The *p*-value of less than 0.05 (*) or 0.01 (**) indicates a significant difference in the data.

## 3. Results

### 3.1. Effect of ε-PL Treatment on the Fruit Disease Index

**Figure 1:**
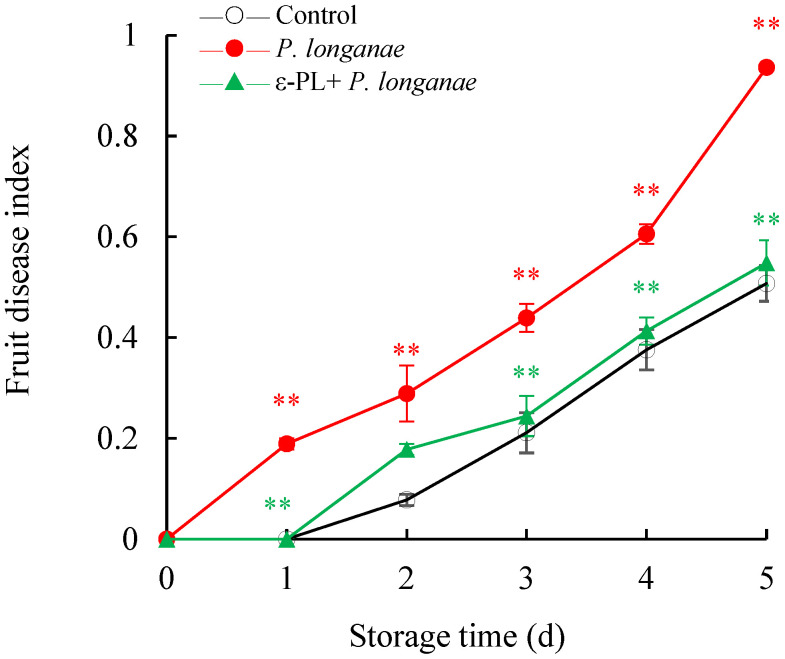
Effects of ε-PL treatment on fruit disease index of *P. longanae*-inoculated longans. Value presented in figure equals mean ± standard error of triplicate analyses, vertical bars express the standard error of mean (*n* = 3). The mark ** represents the significant difference (*p* < 0.05 or *p* < 0.01) between the *P. longanae*-inoculated longans and the control longans on each storage day. The mark ** represents the significant difference (*p* < 0.05 or *p* < 0.01) between the ε-PL-treated *P. longanae*-inoculated longans and the *P. longanae*-inoculated longans on each storage day. ○, Control; ●, *P. longanae*; ▲, ε-PL + *P. longanae*.

The fruit disease index increased in all longan samples at storage time (Figure 1). Longan fruit infected with *P*. *longanae* showed a significantly higher (*p* < 0.01) fruit disease index than the control during the storage period. Compared to infected fruits, ε-PL treatment inhibited the fruit disease index and showed significant effects (*p* < 0.01) at 1 and 3–5 d. Especially on day 5, with the treatment of ε-PL, the fruit disease index was decreased by 41.44% in the *P*. *longanae*-inoculated longans.

### 3.2. Effect of ε-PL treatment on ADP, AMP, ATP, and Energy Charge

**Figure 2:**
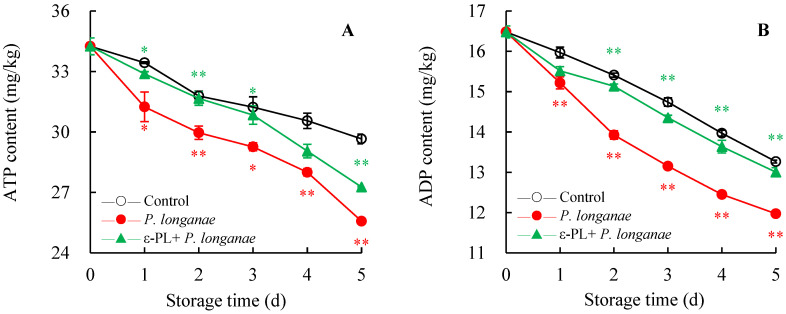

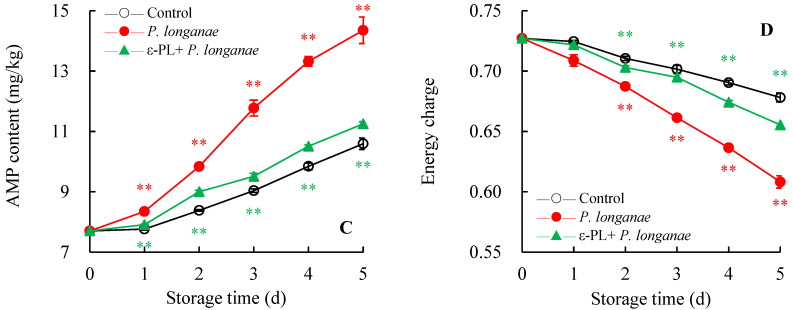
Effects of ε-PL treatment on activities of ATP (**A**), ADP (**B**), and AMP (**C**) contents and energy charge (**D**) in pericarp of *P. longanae*-inoculated longans. Value presented in figure equals mean ± standard error of triplicate analyses, vertical bars express the standard error of mean (*n* = 3). The mark * or ** represents the significant difference (*p* < 0.05 or *p* < 0.01) between the *P. longanae*-inoculated longans and the control longans on each storage day. The mark * or ** represents the significant difference (*p* < 0.05 or *p* < 0.01) between the ε-PL-treated *P. longanae*-inoculated longans and the *P. longanae*-inoculated longans on each storage day. ○, Control; ●, *P. longanae*; ▲, ε-PL + *P. longanae*.

The ATP content decreased during storage, which was related to the dissipation of energy in postharvest fruits. Figure 2A demonstrated that the pericarp ATP content of *P*. *longanae*-inoculated longans was significantly lower (*p* < 0.05) than that in the control group during storage time. The ATP content of ε-PL + *P. longanae*-treated longans was notably higher (*p* < 0.01) than *P*. *longanae*-inoculated longans on day 2 and day 5.

The ADP content (Figure 2B) and energy charge (Figure 2D) showed a similar trend as the ATP content (Figure 2A). The ADP content and energy charge of *P*. *longanae*-inoculated longans exhibited a rapid decline (*p* < 0.01) in the period of 2–5 d storage. However, ε-PL + *P. longanae*-treated longans maintained markedly higher (*p* < 0.01) levels of ADP and energy charge than *P*. *longanae*-inoculated longans at 2–5 d.

As displayed in Figure 2C, the AMP content increased with the storage period, and the highest content of AMP was obtained in *P*. *longanae*-inoculated longans. After being stored for 1–5 days, ε-PL + *P. longanae*-treated longans contained a lower AMP content (*p* < 0.01) than *P*. *longanae*-inoculated longans.

### 3.3. Effect of ε-PL Treatment on Ca^2+^-ATPase Activity

**Figure 3:**
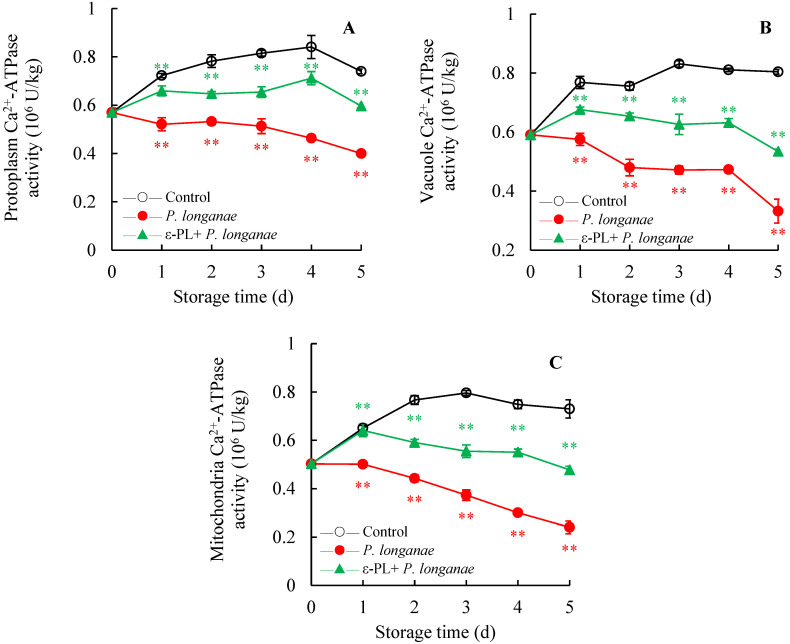
Effects of ε-PL treatment on activities of Ca^2+^-ATPase in membranes of protoplasm (**A**), vacuole (**B**), and mitochondria (**C**) in pericarp of *P. longanae*-inoculated longans. Value presented in figure equals mean ± standard error of triplicate analyses, vertical bars express the standard error of mean (*n* = 3). The mark ** represents the significant difference (*p* < 0.05 or *p* < 0.01) between the *P. longanae*-inoculated longans and the control longans on each storage day. The mark ** represents the significant difference (*p* < 0.05 or *p* < 0.01) between the ε-PL-treated *P. longanae*-inoculated longans and the *P. longanae*-inoculated longans on each storage day. ○, Control; ●, *P. longanae*; ▲, ε-PL + *P. longanae*.

Figure 3 demonstrates that the activity of Ca^2+^-ATPase in mitochondria, protoplasm, and vacuole membrane of the control longans grew during the earlier periods of storage and then decreased. During the whole storage time, the Ca^2+^-ATPase activity of *P*. *longanae*-inoculated longans was markedly lower (*p* < 0.01) than that of the control. However, ε-PL + *P. longanae*-treated longans kept higher Ca^2+^-ATPase activities, and it was notably higher (*p* < 0.01) than *P*. *longanae*-inoculated longans after the same time storage.

### 3.4. Effect of ε-PL Treatment on Mg^2+^-ATPase Activity

**Figure 4:**
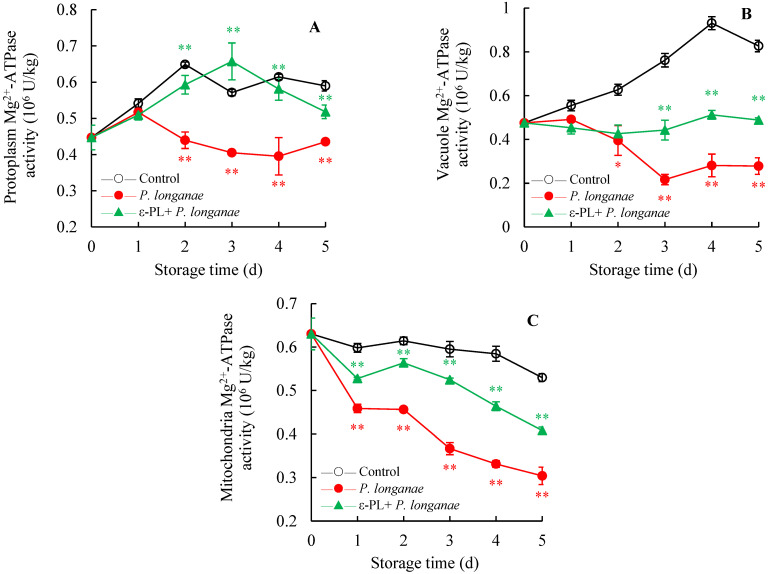
Effects of ε-PL treatment on activities of Mg^2+^-ATPase in membranes of protoplasm (**A**), vacuole (**B**), and mitochondria (**C**) in pericarp of *P. longanae*-inoculated longans. Value presented in figure equals mean ± standard error of triplicate analyses, vertical bars express the standard error of mean (*n* = 3). The mark * or ** represents the significant difference (*p* < 0.05 or *p* < 0.01) between the *P. longanae*-inoculated longans and the control longans on each storage day. The mark ** represents the significant difference (*p* < 0.05 or *p* < 0.01) between the ε-PL-treated *P. longanae*-inoculated longans and the *P. longanae*-inoculated longans on each storage day. ○, Control; ●, *P. longanae*; ▲, ε-PL + *P. longanae*.

At 3–5 d of storage, compared to the control logans, a significantly lower Mg^2+^-ATPase activity (*p* < 0.01) was observed in the protoplasm and vacuole membrane of *P. longanae*-inoculated longans (Figure 4A,B). However, at the same storage time, ε-PL treatment significantly (*p* < 0.01) increased the Mg^2+^-ATPase activity of *P*. *longanae*-inoculated longans (Figure 4A,B). As displayed in Figure 4C, at 1 to 5 days of storage, the Mg^2+^-ATPase activity in mitochondria was significantly reduced (*p* < 0.01) in *P*. *longanae*-inoculated group compared to the control longans. Moreover, within the whole storage time, a significantly (*p* < 0.01) higher level of Mg^2+^-ATPase activity in mitochondria was observed in ε-PL + *P. longanae*-treated longans compared to *P*. *longanae*-infected longans.

### 3.5. Effect of ε-PL Treatment on H^+^-ATPase Activity

**Figure 5:**
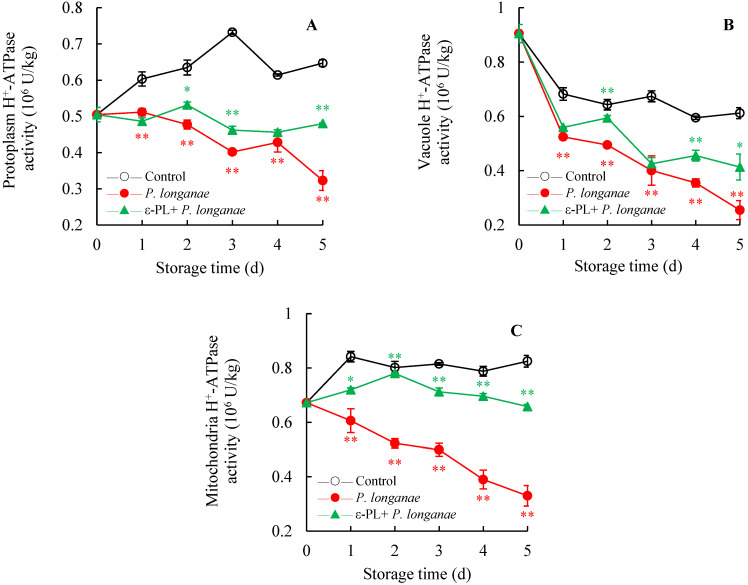
Effects of ε-PL treatment on activities of H^+^-ATPase in membranes of protoplasm (**A**), vacuole (**B**), and mitochondria (**C**) in pericarp of *P. longanae*-inoculated longans. Value presented in figure equals mean ± standard error of triplicate analyses, vertical bars express the standard error of mean (*n* = 3). The mark ** represents the significant difference (*p* < 0.05 or *p* < 0.01) between the *P. longanae*-inoculated longans and the control longans on each storage day. The mark * or ** represents the significant difference (*p* < 0.05 or *p* < 0.01) between the ε-PL-treated *P. longanae*-inoculated longans and the *P. longanae*-inoculated longans on each storage day. ○, Control; ●, *P. longanae*; ▲, ε-PL + *P. longanae*.

Figure 5 illustrates that inoculation with *P*. *longanae* significantly decreased (*p* < 0.01) the activity of H^+^-ATPase in the membranes of mitochondria, protoplasm, and vacuole at 1–5 d. Compared to the longan infected with *P. longanae*, a significantly higher (*p* < 0.01) activity of H^+^-ATPase was observed in the mitochondria membrane of the ε-PL + *P. longanae*-treated longans at 2 to 5 days.

## 4. Discussion

The production of ATP by mitochondria is a critical energy source for the activities of plant life and resistance to pathogenic infection [32,33]. ATP is involved in the synthesis of various essential substances and, in addition, has an important role in maintaining the self-healing capacity and structural integrity of the cell membranes. When plants are infected by pathogenic microorganisms, they will produce a series of defensive reactions, synthesize disease-resistant substances such as phytoalexin and phenolic compounds, resulting in a decrease in energy status [11]. On the other hand, the lack of ATP weakens the normal function of the cell membranes, including the energy synthesis capacity of mitochondria, and then disrupts the cell membranes’ structure integrity [34,35]. In view of this, insufficient energy supply of plant cells will lead to a decline in the ability of plants to synthesize anti-disease substances and resist infection by pathogens, and eventually lead to the occurrence of diseases.

ATPases (covering H^+^-, Mg^2+^-, and Ca^2+^-ATPase) are present mainly in the membranes of mitochondria, protoplasm, and vacuole. ATPases are involved in the hydrolysis of ATP to maintain the normal energy supply in plants [9,36]. Meanwhile, ATPases play a central role in intercellular material transport and signal transmission [36,37,38]. In plants, Ca^2+^-ATPase can hydrolyze ATP to provide energy for intercellular Ca^2+^ transport, while Mg^2+^-ATPase can act synergistically with Ca^2+^-ATPase to maintain cellular osmolarity and prevent peroxidation from damaging cell membranes [3,36,39]. H^+^-ATPase could promote ATP hydrolysis and release energy, provide the energy basis for transmembrane dynamics, and maintain the pH of cellular tissues [36,40]. A decrease in ATPase activity could affect the ion homeostasis in plants, disrupt the integrity of mitochondria, plasma, and vesicle membrane structure, and further cause energy deficit and reduce disease resistance [30,39]. Therefore, intracellular Mg^2+^, H^+^, and Ca^2+^, regulated by ATPase, play primary roles in maintaining functions and integrity of mitochondria and the homeostasis of cellular energy.

The inhibition of Ca^2+^-ATPase activity transiently augments the concentration of cytosolic Ca^2+^, leading to the reduction in or absence of disease resistance [9]. Furthermore, high cytosolic Ca^2+^ concentrations can trigger the formation of *β*-1,4 glucan synthase and callose, which can increase fruit disease resistance [9]. Accompanied by the rapid decline in Ca^2+^-ATPase activity, *Monilinia fructicola* infection accelerated disease progress in peach fruit. However, NO + *M. fructicola* remained a higher level of Ca^2+^-ATPase activity, significantly reducing the incidence and severity of peach fruit disease [9]. In addition, Guo et al. (2018) showed that the activities of H^+^- and Ca^2+^-ATPase, and the content of ATP gradually dropped along with the senescence of strawberry fruits [13]. DNP was an uncoupling agent of the respiratory chain that inhibits ATP production. Compared with the control, a higher decay rate was achieved in the DNP-treated group. Nevertheless, ATP treatment reduced the decrease in H^+^- and Ca^2+^-ATPase activities, and energy charge. The ATP-treated samples had the lowest decay rate [13]. Chen et al. (2018) demonstrated that *P. longanae* infection exacerbated the disease development in harvested longan fruit. The reason was attributed to the infection-induced energy deficit, reflected as lower ATP and ADP levels, lower energy charge, and lower Ca^2+^-, Mg^2+^- and H^+^-ATPase activities that could disorder the transport and distribution of ions, damage the function and structure of mitochondria and vacuole [30]. However, exogenous ATP supply can help maintain the cell membranes’ integrity and better disease resistance in harvested fruit [41,42]. Therefore, it was inferred that the regulation of energy state and ATPase could enhance fruit disease resistance and reduce fruit decay.

**Figure 6:**
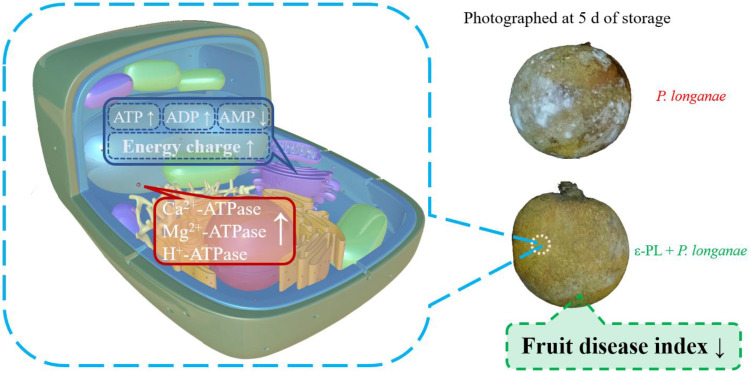
The possible mechanism of ε-PL reduces the incidence of *P. longanae* infection in harvested longan fruit via acting on energy metabolism.

In this study, compared to infected fruits, the ε-PL-treated *P. longanae*-inoculated longans exhibited a prominently lower fruit disease index (Figure 1). In addition, ε-PL + *P. longanae*-treated longans also displayed higher levels of ATP, ADP, and energy charge (Figure 2A,B,D), a lower content of AMP (Figure 2C), and higher H^+^-, Mg^2+^, and Ca^2+^-ATPase activities in mitochondria, protoplasm, and vacuole (Figure 3, Figure 4 and Figure 5). ε-PL was used to prevent *P. longanae* infection, and the corresponding possible mechanism might be that the ε-PL treatment maintained a higher ATP content and energy charge, and also kept higher levels of the ATPase activities to retard ion disorder and protect the function and integrity of the cellular biofilm system (Figure 6).

## 5. Conclusions

In summary, the inhibitor impact of ε-PL treatment on the development of fruit disease might be attributed to the retained higher energy level and ATPase activity, and then the ability of longans against *P. longanae* could be enhanced. These results confirmed that ε-PL treatment has great potential as a practical postharvest processing tool for reducing longans disease during storage.

## Data Availability

The datasets generated for this study are available on request to the corresponding author.
